# INNOVATIONS IN AGING: CREATING A TRANSDISCIPLINARY RESEARCH COLLABORATIVE GRADUATE FELLOWSHIP PROGRAM

**DOI:** 10.1093/geroni/igac059.726

**Published:** 2022-12-20

**Authors:** Diane Martin, Nia Latimer, Katherine Bowers

## Abstract

Social isolation impacts nearly one-quarter of community dwelling adults aged 65+ in the United States. It is associated with poor physical and mental health, cognitive decline, higher healthcare costs, and early mortality. Older adults are at greater risk because they are more likely to live alone, experience a shrinking social network from loss of family and friends, and encounter limitations with driving and mobility resulting from chronic health conditions and sensory impairments. Social isolation in later life is not new; however, the COVID-19 pandemic brought to light the increased risk for negative outcomes. Reducing social isolation is a priority area for our state’s Department of Aging (DoA), and the updated State Plan on Aging highlights the need for innovative approaches to develop and strengthen initiatives addressing social isolation among older adults. The DoA is advancing multiple projects, including a partnership with our public university to create a transdisciplinary graduate fellowship program. The program brought together professional students from medicine, nursing, pharmacy, and social work and resulted in a community of practice in which fellows engaged with DoA and university faculty to share best practices and receive training in transdisciplinary research. In this session, the first presentation will focus on development of the fellowship program and highlight the success of the inaugural year; the second session will focus on the future goals of the fellowship program, and the third presentation will share how we plan to expand the partnership between our public university and our state’s Department of Aging.

